# Supplementary material for: Factors associated with the use of diet and the use of exercise for prostate cancer by long-term survivors
Source: PLoS One. 2019 Oct 3;14(10):e0223407. doi: 10.1371/journal.pone.0223407 (PMC6776329; doi:10.1371/journal.pone.0223407)
Supplement: S1 Table — (DOCX) [file pone.0223407.s001.docx]

**S1 Table. Reasons for ─ and sources of information on ─ current use of diet and exercise for prostate cancer and/or its treatment side effects**

|  | **Diet (current use)** | **Exercise (current use)** |
| --- | --- | --- |
|  |  |  |
|  |  |  |
|  | **n=118** | **n=78** |
|  | **n (%)** | **n (%)** |
| **Reasons for current use^‡^:** |  |  |
| To boost my immune system | 54 (45.8) | 16 (20.5) |
| To prevent cancer returning or spreading | 54 (45.8) | 9 (11.5) |
| To deal with the side effects of treatment | 20 (17.0) | 27 (34.6) |
| To make me feel better | 53 (44.9) | 45 (57.7) |
| To cure the cancer | 7 (5.9) | 2 (2.6) |
| To cope with the stress | 7 (5.9) | 16 (20.5) |
| To provide hope | 13 (11.0) | 3 (3.9) |
| To do as much as I can for myself | 54 (45.8) | 26 (33.3) |
| Because they are not toxic | 7 (5.9) | 1 (1.3) |
| Because I am disappointed in the other treatments | 3 (2.5) | 2 (2.6) |
| Other | 8 (6.8) | 7 (9.0) |
| Not answered | 9 (7.6) | 6 (7.7) |
| **Sources of information on diet and exercise^‡^:** |  |  |
| Other men with prostate cancer | 14 (11.9) | 1 (1.3) |
| Friends or relatives | 28 (23.7) | 12 (15.4) |
| Newspaper, mags, radio TV | 50 (42.4) | 12 (15.4) |
| Internet | 12 (10.2) | 2 (2.6) |
| Doctor | 40 (33.9) | 20 (25.6) |
| Religious practice | 0 (0) | 0 (0) |
| Naturopath/herbalist | 6 (5.1) | 0 (0) |
| Books | 5 (4.2) | 0 (0) |
| Allied health | 5 (4.2) | 3 (3.9) |
| Other | 15 (12.7) | 18 (23.1) |
| Not answered | 9 (7.6) | 25 (32.1) |

‡ Multiple response question; percentages may add to more than 100%
