# Supplementary material for: Factors associated with the use of diet and the use of exercise for prostate cancer by long-term survivors
Source: PLoS One. 2019 Oct 3;14(10):e0223407. doi: 10.1371/journal.pone.0223407 (PMC6776329; doi:10.1371/journal.pone.0223407)
Supplement: S2 Table — (DOCX) [file pone.0223407.s002.docx]

**S2 Table. Estimated prevalences of current diet and exercise changes with and without multiple imputation of missing data for surviving participants who completed the baseline but not the 10-year questionnaire, and for participants who completed the 10-year questionnaire but had missing data for one or more variables**

|  | **Ever** | **Currently** |
| --- | --- | --- |
| **Diet and exercise changes for prostate cancer and/or treatment side effects^** | **% (95% CI)** | **% (95% CI)** |
| **Diet changes:** |  |  |
| Original analysis (n=996)^1^ | 13.5% (11.3-15.6) | 11.8% (9.8-13.9) |
| Multiple imputation analysis (n=1634)^2^ | 15.9% (13.2-18.6) | 13.3% (11.0-15.6) |
| **Exercise changes:** |  |  |
| Original analysis (n=996)^1^ | 10.2% (8.4-12.1) | 7.8% (6.2-9.5) |
| Multiple imputation analysis (n=1634)^2^ | 12.1% (9.8-14.4) | 8.9% (7.0-10.8) |

^1^Prevalence estimates from original analysis of 996 participants redisplayed here for convenience.

^2^Prevalence estimates derived after multiple imputation of missing data for the 638 participants who completed the baseline questionnaire, but not the 10-year questionnaire, and for participants who completed the 10-year questionnaire but had missing data for one or more variables. Values of missing data were imputed 100 times using the method of chained equations. Variables in the imputation models were the dependent and independent variables in the original multivariate regression models plus baseline socio-economic status of place of residence, place of residence, employment status, health insurance and marital status. Age, education, and country of birth at 10-year follow-up were estimated directly from corresponding baseline values. Imputation and analysis of imputed data were performed using the mi suite of commands in Stata version 14.
