# Supplementary material for: Factors associated with the use of diet and the use of exercise for prostate cancer by long-term survivors
Source: PLoS One. 2019 Oct 3;14(10):e0223407. doi: 10.1371/journal.pone.0223407 (PMC6776329; doi:10.1371/journal.pone.0223407)
Supplement: S5 Table — (DOCX) [file pone.0223407.s005.docx]

**S5 Table. Demographic and clinical characteristics of PCOS men invited to participate in the 10-year questionnaire; respondents versus non-respondents**

|  | **PCOS 10-year survey** | |
| --- | --- | --- |
| **Characteristic** | **Respondents† n (%)** | **Non-respondents or PCOS men lost to follow-up† n (%)** |
|  | **996 (100.0)** | **638 (100.0)** |
| **Age ^** |  |  |
| <65 | 164 (16.5) | 84 (13.2) |
| 65-69 | 267 (26.8) | 143 (22.4) |
| 70-74 | 299 (30.0) | 181 (28.4) |
| 75+ | 266 (26.7) | 230 (36.1) |
| **Education** |  |  |
| University or college degree | 297 (29.8) | 150 (23.5) |
| High school | 670 (67.3) | 446 (69.9) |
| Less than high school | 25 (2.5) | 41 (6.4) |
| Missing | 4 (0.4) | 1 (0.2) |
| **Socio-economic status of residence area at time of diagnosis** | |  |
| 1- Highest SES | 387 (38.9) | 222 (34.8) |
| 2 | 188 (18.9) | 126 (19.7) |
| 3 | 204 (20.5) | 123 (19.3) |
| 4 | 140 (14.1) | 99 (15.5) |
| 5- Lowest SES | 73 (7.3) | 66 (10.3) |
| Missing | 4 (0.4) | 2 (0.3) |
| **Place of residence at time of diagnosis** |  |  |
| Major city | 687 (69.0) | 442 (69.3) |
| Inner regional | 238 (23.9) | 144 (22.6) |
| Outer regional/ remote/ very remote | 68 (6.8) | 50 (7.8) |
| Missing | 3 (0.3) | 2 (0.3) |
| **Country of birth** |  |  |
| In Australia | 768 (77.1) | 464 (72.7) |
| In another country | 227 (22.8) | 173 (27.1) |
| Missing | 1 (0.1) | 1 (0.2) |
| **Overall cancer severity at diagnosis ^^** |  |  |
| Localised low risk | 341 (34.2) | 197 (30.9) |
| Localised intermediate risk | 359 (36.0) | 191 (29.9) |
| Localised high risk | 176 (17.7) | 132 (20.7) |
| Stage T3-4 | 68 (6.8) | 43 (6.7) |
| Unknown | 52 (5.2) | 75 (11.8) |
| **First treatment after diagnosis** |  |  |
| Active Surveillance | 99 (9.9) | 79 (12.4) |
| Androgen Deprivation Therapy | 25 (2.5) | 21 (3.3) |
| Combined EBRT/ADT | 89 (8.9) | 66 (10.3) |
| External Beam Radiotherapy | 62 (6.2) | 43 (6.7) |
| HDR Brachytherapy | 48 (4.8) | 11 (1.7) |
| LDR Brachytherapy | 40 (4.0) | 13 (2.0) |
| Orchidectomy | 0 (0.0) | 3 (0.5) |
| Radical Prostatectomy | 608 (61.0) | 349 (54.7) |
| Missing | 25 (2.5) | 53 (8.3) |

† Respondents are men who completed the 10-year survey; Non-respondents are living men in the PCOS cohort remaining in the study as of January 2011, who were invited but did not complete the 10-year questionnaire; PCOS men lost follow-up includes PCOS participants who, as of January 2011, had withdrawn from PCOS or were no longer contactable; ^ Age represents age at date of 10-year questionnaire completion for respondents, and age at median date of 10-year questionnaire completion for non-respondents and PCOS men lost follow-up;**^^** Localised (stage 1 or 2) risk groups- low risk (PSA≤10, Gleason score ≤6, and clinical stage=T1-2a), intermediate risk (10<PSA≤20, Gleason score=7 or clinical stage=T2b) high-risk (PSA >20, Gleason score>7, or clinical stage T2c);
