# Supplementary material for: Factors associated with the use of diet and the use of exercise for prostate cancer by long-term survivors
Source: PLoS One. 2019 Oct 3;14(10):e0223407. doi: 10.1371/journal.pone.0223407 (PMC6776329; doi:10.1371/journal.pone.0223407)
Supplement: S1 Fig — (DOCX) [file pone.0223407.s006.docx]

Potentially eligible localised and advanced prostate cancer cases (n=3195)

Patients who gave consent (n=2031)

Withdrew from PCOS before baseline (n=36)

Non-consenters (n=1164):

- Refused consent by doctor (n=537)
- Refused consent by patient (n=627)

**Patients who completed 10-year questionnaire (n=996)**

Patients invited to participate in 10-year questionnaire (n=1427)

Did not complete 10-year questionnaire (n=431)

Died before Jan. 2011 (n=361)

Died (n=358) or lost to follow-up (n=207) before 10-year survey

**Patients still alive in Jan. 2011 (n=1634)**

Lost to follow-up before Jan. 2011 (n=207)

Died (n=358) or lost to follow-up (n=207) before 10-year survey

Patients assessed at baseline between Oct. 2000 and Oct. 2002 (n=1995)

**S1 Fig. Flow diagram showing patients’ participation and follow-up**
