# Supplementary material for: Factors associated with the use of diet and the use of exercise for prostate cancer by long-term survivors
Source: PLoS One. 2019 Oct 3;14(10):e0223407. doi: 10.1371/journal.pone.0223407 (PMC6776329; doi:10.1371/journal.pone.0223407)
Supplement: Attachment — Submitted filename: Responses to Reviewers.docx [file pone.0223407.s009.docx]

**Academic editor:**

***1. When submitting your revision, we need you to address these additional requirements.***

**A.1	*Please ensure that your manuscript meets PLOS ONE's style requirements, including those for file naming. The PLOS ONE style templates can be found at***

[***http://www.journals.plos.org/plosone/s/file?id=wjVg/PLOSOne_formatting_sample_main_body.pdf***](http://www.journals.plos.org/plosone/s/file?id=wjVg/PLOSOne_formatting_sample_main_body.pdf) ***and***

[***http://www.journals.plos.org/plosone/s/file?id=ba62/PLOSOne_formatting_sample_title_authors_affiliations.pdf***](http://www.journals.plos.org/plosone/s/file?id=ba62/PLOSOne_formatting_sample_title_authors_affiliations.pdf)

We believe our manuscript now meets PLOS ONE's style requirements.

**A.2	*2. In the Methods, please clarify whether participants provided informed consent to participate, and if so whether consent was written or verbal.***

This has been added to the methods section (page 6, lines 101-104).

**A.3	*3.  We note that you have indicated that data from this study are available upon request. PLOS only allows data to be available upon request if there are legal or ethical restrictions on sharing data publicly. For information on unacceptable data access restrictions, please see*** [***http://journals.plos.org/plosone/s/data-availability#loc-unacceptable-data-access-restrictions***](http://journals.plos.org/plosone/s/data-availability#loc-unacceptable-data-access-restrictions)***.***

***a) If there are ethical or legal restrictions on sharing a de-identified data set, please explain them in detail (e.g., data contain potentially identifying or sensitive patient information) and who has imposed them (e.g., an ethics committee). Please also provide contact information for a data access committee, ethics committee, or other institutional body to which data requests may be sent.***

***b) If there are no restrictions, please upload the minimal anonymized data set necessary to replicate your study findings as either Supporting Information files or to a stable, public repository and provide us with the relevant URLs, DOIs, or accession numbers. Please see*** [***http://www.bmj.com/content/340/bmj.c181.long***](http://www.bmj.com/content/340/bmj.c181.long) ***for guidelines on how to de-identify and prepare clinical data for publication. For a list of acceptable repositories, please see*** [***http://journals.plos.org/plosone/s/data-availability#loc-recommended-repositories***](http://journals.plos.org/plosone/s/data-availability#loc-recommended-repositories)***.***

***We will update your Data Availability statement on your behalf to reflect the information you provide.***

We have updated our data availability statement as follows:

Data used in this study are partially restricted for use because of restrictions imposed by ethical approval, the research aims governing the study and consent agreements with study participants. The data is available through an application to the Cancer Council NSW Cancer Research Division and with approval from the Cancer Council NSW Ethics Committee. Researchers who wish to access data owned or held by Cancer Council NSW must hold all relevant ethics approvals before access can be granted. Researchers may be required to enter into a Data Transfer Agreement. To find out how to access data please contact the Cancer Research Division at [research@nswcc.org.au](mailto:research@nswcc.org.au). Details and contact information are available at [https://www.cancercouncil.com.au/research/for- researchers/ethics/](https://www.cancercouncil.com.au/research/for-%20researchers/ethics/). The data custodian for the NSW Prostate Cancer Care and Outcomes Study is Cancer Council NSW.

**A.5	*4. We note that you have included the phrase “data not shown” in your manuscript. Unfortunately, this does not meet our data sharing requirements. PLOS does not permit references to inaccessible data. We require that authors provide all relevant data within the paper, Supporting Information files, or in an acceptable, public repository. Please add a citation to support this phrase or upload the data that corresponds with these findings to a stable repository (such as Figshare or Dryad) and provide and URLs, DOIs, or accession numbers that may be used to access these data. Or, if the data are not a core part of the research being presented in your study, we ask that you remove the phrase that refers to these data.***

By “data not shown” we did not mean to imply this result comes from “inaccessible data”. We meant that the result (a single percentage) is not displayed in the tables or figures. The result still comes from the primary data set which is available under the conditions described in our data availability statement. Hence we have removed the phrase “data not shown” from the text (page 17, line 335).

**A.6	*5. Thank you for stating the following in your Competing Interests section:***

***[No].***

**** Please complete your Competing Interests on the online submission form to state any Competing Interests. If you have no competing interests, please state "The authors have declared that no competing interests exist.", as detailed online in our guide for authors at*** [***http://journals.plos.org/plosone/s/submit-now***](http://journals.plos.org/plosone/s/submit-now)

**** This information should be included in your cover letter; we will change the online submission form on your behalf.***

We have modified the Competing Interests section in the manuscript on page 22, lines 448-449 to say: "The authors have declared that no competing interests exist."

**A.7	*6. Please amend either the title on the online submission form (via Edit Submission) or the title in the manuscript so that they are identical.***

The title on the online submission form and the manuscript appear to be identical (Factors associated with the use of diet and the use of exercise for prostate cancer by long-term survivors).

**Reviewer #1:**

***In this cross-sectional analysis, the investigators aimed to (1) describe the prevalence of self-reported diet and exercise changes among prostate cancer survivors and (2) examine associations with sociodemographic, clinical, HRQOL, and psychological factors. While these are clinically important aims that could help to inform targeted lifestyle interventions among men diagnosed with prostate cancer, the significance and soundness of this study are limited by its design.***

***Major comments:***

**1.1	*• Selection bias is likely given that the analyses were restricted to individuals who survived and remained under follow-up long enough to return a 10-year follow-up questionnaire.***

What the reviewer calls “selection bias”, we consider to be “non-response bias” as described on page 19, lines 377-380, where we addressed this as a limitation as follows:

Second, a potentially important source of bias is the non-response of 39% of surviving PCOS participants to the 10-year questionnaire. Somewhat reassuringly, however, is the fact that other than a higher proportion of non-respondents being aged 75 years and over, respondents and non-respondents had similar demographic and clinical characteristics [S5 Table].

While we agree with the reviewer that non-response of ***surviving*** participants can be an important source of bias, we don’t believe that our inability to survey ***deceased*** participants is a source of bias. That is, because our aim was to estimate exercise and dietary use of ***surviving*** long-term prostate cancer survivors (often called a mortal cohort analysis), inferences involving deceased participants (ie an immortal cohort analysis) are not appropriate for our aims.

Nonetheless, we do accept that attrition of surviving participants is a potentially important source of bias for most long-term longitudinal studies such as ours. Hence, in light of the reviewer’s comment we have now performed the following sensitivity analysis described on pages 10-11, lines 212-222:

Sensitivity analyses were conducted to assess whether estimates were unduly affected by missing data. In these analyses, multiple imputation was used to impute missing 10-year data for the 638 surviving PCOS participants who did not complete the 10-year questionnaire. Missing data were also multiply imputed for participants who completed the 10-year questionnaire but had missing data for one or more variables. Data were imputed 100 times using the method of chained equations [37]. Variables in the imputation models were the dependent and independent variables in the original multivariable regression models plus baseline socio-economic status of place of residence, place of residence, employment status, health insurance and marital status (age, education, and country of birth at the 10-year follow-up were estimated directly from corresponding baseline values). The combined imputed and original data were then analysed using estimation techniques for multiply imputed data.

Results of the multiple imputation analyses are now shown in S2-S4 Tables and S2-S3 Figs and are described on page 15, lines 286-289 as follows:

Analyses using the multiply-imputed data produced marginally, but not materially, higher estimates for prevalences of current diet and exercise use (S2 Table). The estimated magnitudes of associations from the original analyses were not materially different from those obtained from analyses using the multiply-imputed data (S2-S4 Tables and S2-S4 Figs).

The Limitations section of the Discussion on page 19, lines 376-381 (and referred to above) has been modified to refer to the multiple imputation analyses as follows:

Second, a potentially important source of bias is the non-response of 39% of surviving PCOS participants to the 10-year questionnaire. Somewhat reassuringly, however, is the fact that other than a higher proportion of non-respondents being aged 75 years and over, respondents and non-respondents had similar demographic and clinical characteristics [S2 Table]. Moreover, the inclusion of the 10-year non-respondents through multiple imputation made little difference to the original estimates (S2-S4 Tables and S1-S2 Figs) or to our overall conclusions.

**1.2	*• Measurement bias is likely as men recalled whether they had ever changed certain behaviors (e.g., increased fruit intake, increased walking) over the past 10-year period.***

We accept that men might struggle to remember whether they had “ever” made changes to their diet or exercise over the previous 10-year period. However, our primary outcomes which we assessed in the multivariable analyses relate to “currently” eating and exercising differently (which would not be vulnerable to recall error). Nonetheless, in light of the reviewer’s comment, we have added the following to the discussion on limitations on page 19, lines 384-387:

Fourth, self-report of having “ever” made changes to diet or exercise for prostate cancer is vulnerable to recall error as men might struggle to remember changes over the 10-year post-diagnosis period. However, self-report of “current” diet or exercise use for prostate cancer would not be vulnerable to the same recall error.

***Minor comments:***

**1.3	*• The composite measures of “change in diet and exercise” do not capture information on specific behaviors, which would be important to inform targeted interventions.***

Table 2 shows the specific diet changes irrespective of exercise changes, and exercise changes irrespective of diet changes. So the only specific changes not shown in Table 2 are combinations of exercise and diet changes for men who change both diet and exercise. The reason for this is that so few men changed both diet and exercise (4.3% “ever” and 3.8% “currently”) and the specific combinations are used by less than 1% of men (which was the criterion for listing specific changes in Table 2, as stated in the footnote below the table).

**1.4	*• The restriction to “change to help with prostate cancer” does not capture positive changes that may be made to benefit other clinically relevant outcomes in this patient population, such as cardiovascular disease.***

We chose to focus on diet and exercise changes that survivors make to help with their prostate cancer because we believe they are more interesting outcomes than changes made for health and well-being more generally. More specifically, we chose our outcomes because they capture not only men’s willingness to make healthy lifestyle changes, but also men’s awareness that such changes may help them with their prostate cancer. Nonetheless, the reviewer makes a good point that diet and exercise changes made improving health and well-being may “help with their prostate cancer”, even if that was not the intention of the survivor. Hence, we have added the following text on page 17, lines 332-341:

In our study, for example, only 15.9% of survivors reported currently eating, exercising or doing both differently to help with their prostate cancer and/or its treatments’ side effects (Table 2), yet 51.4% of survivors reported currently eating, exercising or doing both differently for any reason related to improving health and well-being (including to help with prostate cancer) . In the current study, we chose to focus on diet and exercise changes survivors make to help with their prostate cancer because these outcomes capture both men’s willingness to make healthy lifestyle changes and also their awareness that such changes might help with their prostate cancer. It is important to note, however, that although many survivors in our cohort reported changing eating and exercise habits for reasons unrelated to their cancer, such habits may also help them with their cancer.

**1.5	*• The concluding remarks about CAM do not seem central to the a priori aims.***

The reason we discuss CAM use in this paper is because the study’s original aim was to look at CAM, diet and exercise use for prostate cancer. However, when we submitted the original paper (1) in which we reported all activities together, one reviewer suggested (and the PLOS ONE editor agreed) that we focus only on CAMs or only on diet/exercise because there was too much information for one paper. Consequently, our reporting of the study’s results has been divided into two papers. So although CAM use is not central to the aims of this particular paper, we think it is important to discuss and compare the findings reported in our two papers, particularly given the credible evidence supporting the use of diet and exercise, and the absence of evidence supporting the use of CAMs.

Nonetheless, we accept the reviewer’s point that our discussion of CAM use may have seemed out of context. Hence, we have added the following text on page 5, lines 90-93:

We also discuss the cohort’s use of diet and exercise for prostate cancer in comparison to their use of complementary and alternative medicines (CAMs) for prostate cancer ─ the latter having been reported in detail in a previous publication [20].

**Reviewer #2:**

**2.1	*(Introduction, Page 4): “There appears to be little or no evidence from RCT’s that changes in physical activity levels improve markers of prostate cancer progression” – This might be true, but there is epidemiological evidence to this effect, pertaining to prostate cancer progression / aggression, and so too post-diagnosis physical activity and overall survival. It may be at-least worthwhile to highlight this, as the working hypothesis isn’t narrowcast to quality of life only – which use patient self-report much like your own current study, thus as dutiful I would think:***

[***https://www.ncbi.nlm.nih.gov/pubmed/26276753***](https://www.ncbi.nlm.nih.gov/pubmed/26276753)
[***https://www.ncbi.nlm.nih.gov/pubmed/21610110***](https://www.ncbi.nlm.nih.gov/pubmed/21610110)
[***https://www.ncbi.nlm.nih.gov/pubmed/21205749***](https://www.ncbi.nlm.nih.gov/pubmed/21205749)

As suggested, we have modified the following text on page 4, lines 65-67 which now cites the three papers listed by the reviewer:

With regards to exercise, there appears to be little or no evidence from RCTs that changes in physical activity levels slow prostate cancer progression, but evidence of this effect has been found in observational studies [12-14].

**2.2	*(Methods, Page 8, Diet and Exercise Changes): Unsure why “or exercise program in a separate question” is bracketed. It is not a lower-order question, and should simply be included in the statement / sentence. Seems to be a second-order issue with the authors more interested in diet. Treat both equally given the title of the paper and outcomes of the paper aim for this.***

It is our intention to give diet and exercise equal weight in this paper. The term “or exercise program in a separate question” was bracketed simply to avoid repeating words common to the diet and exercise questions. However, to avoid the perception that we are more interested in diet, we have expanded our description on page 9, lines 165-176 of the questions men were asked as follows:

Men were asked whether they had “ever” made a change to their diet to help with their prostate cancer and/or its treatments’ side effects. If they answered yes they were then asked whether they were “currently” eating differently to help with their prostate cancer and/or its treatments’ side effects. Participants were asked about their specific dietary changes with available response options being: increased/decreased fruit, soy products, vegetables, dairy, fats, oils, fried foods, processed meats, red meat, dairy products plus free-text fields for user–specified options. Men were also asked whether they had “ever” made a change to the exercise they do to help with their prostate cancer and/or its treatments’ side effects. If they answered yes they were then asked whether they were “currently” exercising differently to help with their prostate cancer and/or its treatments’ side effects. Participants were asked to describe any changes in exercise and these free-text fields were subsequently coded for analysis.

**2.3	*Beyond this, is the question: “have you ever made a change” really sensitive enough? Seems remarkably general. Of interest would be “how long” they upheld the change, because health behaviour change is complex, and sustaining a behaviour change while undergoing treatment is even more difficult that simply indicating whether “a change” was transiently made. Was this asked or measured? Ifso consider adding to the paper as it will have meaning towards behaviour change coaching or plans in this population too.***

When designing the questionnaire, we were aware that questions asking “have you ever made a change” were vulnerable to recall error, given that men needed to remember changes made over a 10 year period. This is one of the reasons that the “ever change” variables are not our primary outcomes (for more on this, please see our response to comment 1.2 above). Questions asking men to recall “how long” were not asked, but presumably would have also been vulnerable to recall error. Studies using repeated questionnaires about diet and exercise over time may be better suited for measuring “how long” men sustain their behavioural changes.

***2.4	(Methods, Page 9, Statistical Methods): It is outlined here that Independent Variables include Age - <65, 65-69, 70-75, 75+ with the age ranging from 52 to 80 – but in your methods and your abstract, you stated men <70 years…. Can you please explain this? Were the Men <70 years that age at recruitment? Thus 10 year follow-up can be up to 80 years of age? Or did this study only include analysis of men currently 70 years or younger? In either case, this might need to be more clearly detailed so as to be less ambiguous.***

As stated in the abstract, men were “aged <70 years *at diagnosis*”. The age groups listed in the methods section and in Table 3 relate to age at completion of the 10-year questionnaire (this is explained in the footnote of Table 3, but we have now also added the text “age at completion of the 10-year questionnaire” to the Methods section).

The reason there are 3 eighty-year-olds who completed the 10-year questionnaire is that these men were 69 at diagnosis and they completed the 10-year questionnaire between 10 and 11 years after diagnosis. As stated on page 5, lines 104-106, the 10-year questionnaire was administered - on average - 10 years after diagnosis, but this ranged between 9 and 12 years after diagnosis. We appreciate the reviewer being highly observant in spotting this rounding anomaly. However we don’t think this requires a detailed explanation in the paper as there is currently sufficient information for readers to work out why there might be some eighty-year-olds who completed the 10-year questionnaire.

***2.5	Was there any reason that you decided to exclude subjects with missing data from psychological/HRQOL domains instead of using missing data imputations?***

As suggested, we have now conducted sensitivity analyses involving multiple imputation of missing data for the 638 surviving PCOS participants who did not complete the 10-year questionnaire, and for participants who completed the 10-year questionnaire but had missing data for one or more variables. Please see our response to comment 1.1 for details.

***2.6	Did this apply for other data or variables?***

To clarify this, we have added the following on page 10, lines 199-202:

Subjects with missing data on any independent variable were excluded from regression analyses, but ‘unknown’ test results for clinical variables were analysed as distinct categories (because ‘unknown’ test results are primarily due to the absence of testing and thus the result is ‘unknown’ to the subject).

We also now report the number of subjects excluded due to missing data in the footnotes below Tables 3 and 4 as follows:

16 of the 996 participants were excluded from regression analyses due to missing data.

***2.7	(Table 2): I am surprised there is no information around resistance exercise. Why was walking judged to be different to aerobic exercise?***

We kept walking as a separate category because we thought it was interesting that it was such a common activity. Nonetheless, to correct the labelling problem pointed out by the reviewer we have relabelled the category “More aerobic exercise” to “More aerobic exercise (other than walking)”.

***2.8	And why has no resistance exercise or muscle building exercise been included? Did the questionnaire assist patients in understanding how different types of exercise or physical activity are defined? If not this is a limitation that must be noted, because participants often miscategorise activities as they do not understand the true definition – it is noticeable in the Godin Leisure-Time Questionnaire often. The exercise portion as a result seems incomplete in this Table – though this may be a consequence of the fact that it was coded by the researchers using free-form entry by participants only.***

The reviewer’s query regarding resistance exercise led us to re-examine our results. We had excluded resistance exercise from the table because 10 and 7 men reported “ever” and “currently” doing resistance exercises to help with their prostate cancer, respectively, and we had (incorrectly) judged that these numbers were not more than 1% of men (ie the rule specified in the footnote for including a category in the table). On reflection, however, n=10 corresponds to 1.004% of the cohort, so this category should have been included. We have now included “resistance exercises” in Table 2 and have made the language regarding our rule for including categories more clear (see below for new Table 2). We thank the reviewer for raising this issue.

With regards to the issue of respondent miscategorisation, this was not possible in our study because unlike the Godin Leisure-Time Questionnaire, our questionnaire did not ask respondents to categorise their activities. That is, our questionnaire asked respondents to describe their changes in exercise in free-text fields and these were subsequently coded by us. So while it is possible that we made errors in classifying some activities (we tried our best not to), it was not possible for respondents to make thee errors.

A similar problem that did arise, however, was that respondents sometimes did not provide as much information as we would have liked to enable us to classify their activity type. In particular, a number of men said that they were currently participating (and/or had ever participated) in gym activities to help with their prostate cancer, but they did not specify what the gym activities were. Consequently, we did not include ‘gym activities’ as a separate category in Table 2 because we did not know exactly what the activities were. But in light of the reviewer’s concern that certain exercise types (such as resistance exercise) may have been underrepresented, we now include “Unspecified gym activities” as a category in Table 2. We then explain in footnotes below the table how this category is likely to have resulted in underestimates for the use of aerobic exercise, pelvic floor exercises and/or resistance training. Table 2 is now as follows:

**Table 2. Types of diet and exercise changes ever and currently used for prostate cancer and/or its treatments’ side effects**

|  | **Ever** | **Currently** |
| --- | --- | --- |
| **Diet and exercise changes for prostate cancer and/or treatments’ side effects^** | **n (% of 996)** | **n (% of 996)** |
| **Changes to diet, exercise or both** | **193 (19.4)** | **158 (15.9)** |
| **Diet changes:** | **134 (13.5)** | **118 (11.8)** |
| More fruit | 86 (8.6) | 79 (7.9) |
| More soy products | 37 (3.7) | 30 (3.0) |
| More vegetables | 91 (9.1) | 87 (8.7) |
| Less dairy | 49 (4.9) | 48 (4.8) |
| Less fats, oils or fried foods | 61 (6.1) | 60 (6.0) |
| Less processed meats | 71 (7.1) | 71 (7.1) |
| Less red meat | 66 (6.6) | 63 (6.3) |
| **Exercise changes:** | **102 (10.2)** | **78 (7.8)** |
| More walking | 44 (4.4) | 40 (4.0) |
| More aerobic exercise (other than walking) | 15 (1.5) | 13 (1.3) |
| Pelvic floor exercises | 33 (3.3) | 19 (1.9) |
| Resistance exercises | 10 (1.0) | 7 (<1%) |
| Unspecified gym activities* | 18 (1.8) | 13 (1.3) |

Categories of specific diet and exercise changes are listed if ever used by over 1% of men.

^ Men may have more than one type of diet and or exercise change

* Unspecified gym activities may include “more aerobic exercise”, “pelvic floor exercises” and/or “resistance exercises” (hence numbers for these categories are likely to be underestimates).

**2.9	*(Discussion, Page 15): It discusses prevalence studies in the 2nd paragraph – yet it seems to have missed this paper:*** [***https://www.ncbi.nlm.nih.gov/pubmed/27647712***](https://www.ncbi.nlm.nih.gov/pubmed/27647712) ***which highlights this type of information pertaining to aerobic guidelines and prostate cancer patients with bone metastases. This might be relevant.***

We thank the reviewer for bringing this paper to our attention and we have added it to the reference list (reference 44). However we haven’t discussed that paper’s prevalence estimates because with only 55 participants, the estimates would be considerably imprecise.

**2.10	*Also, why was the “data not shown” for the Men who listed “changing exercise or diet for any reason”.***

By “data not shown”, we simply meant that the percentage is not displayed in the tables or figures. We have now removed the phrase.

**2.11	*Some of these Men may have changed these factors following their prostate cancer diagnosis as a catalyst for the change even if they do not directly attribute to using it “as medicine”. That is, the Men might not have identified their decision to change exercise or diet habits was to “treat their cancer or side-effects” but they may have changed exercise or diet habits “to be more healthy” following their cancer diagnosis. So, you might be removing legitimately interesting data simply because Men didn’t narrowcast their own responses to a self-report questionnaire 10 years post-diagnosis. This may also need to be a limitation acknowledged in your paper. Men might have improved their lifestyle practices, noting that general health and wellbeing is important, not recognising that actually improving general health and wellbeing while dealing with cancer and treatment will itself help “with their prostate cancer”…. This is a flaw of the questionnaire not clearly being able to delineate between the nuance of this, and not the participants themselves. So, this definitely needs some level of appreciation in your paper, otherwise it makes the situation look far direr than it probably is.***

We chose to focus on diet and exercise changes that survivors make to help with their prostate cancer because we believe they are more interesting outcomes than changes for any reason. More specifically, we chose our outcomes because they capture not only men’s willingness to make healthy lifestyle changes, but also men’s awareness that such changes may help them with their prostate cancer. Nonetheless, the reviewer makes a good point that diet and exercise changes made “for any reason” may “help with their prostate cancer”, even if that was not the intention of the survivor. Hence, we have added the following text on page 17, lines 332-341:

In our study, for example, only 15.9% of survivors reported currently eating, exercising or doing both differently to help with their prostate cancer and/or its treatments’ side effects (Table 2), yet 51.4% of survivors reported currently eating, exercising or doing both differently for any reason related to improving health and well-being (including to help with prostate cancer) . In the current study, we chose to focus on diet and exercise changes survivors make to help with their prostate cancer because these outcomes capture both men’s willingness to make healthy lifestyle changes and also their awareness that such changes might help with their prostate cancer. It is important to note, however, that although many survivors in our cohort reported changing eating and exercise habits for reasons unrelated to their cancer, such changes may also help them with their cancer.
